# Noise Reduction in Arterial Spin Labeling Based Functional Connectivity Using Nuisance Variables

**DOI:** 10.3389/fnins.2016.00371

**Published:** 2016-08-23

**Authors:** Kay Jann, Robert X. Smith, Edgar A. Rios Piedra, Mirella Dapretto, Danny J. J. Wang

**Affiliations:** ^1^Laboratory of FMRI Technology, Department of Neurology, University of California Los AngelesLos Angeles, CA, USA; ^2^Department of Psychiatry and Biobehavioral Sciences, University of California Los AngelesLos Angeles, CA, USA

**Keywords:** functional connectivity (FC), noise reduction, default mode network (DMN), arterial spin labeling (ASL), blood oxygenation level dependent (BOLD), cerebral blood flow (CBF)

## Abstract

Arterial Spin Labeling (ASL) perfusion image series have recently been utilized for functional connectivity (FC) analysis in healthy volunteers and children with autism spectrum disorders (ASD). Noise reduction by using nuisance variables has been shown to be necessary to minimize potential confounding effects of head motion and physiological signals on BOLD based FC analysis. The purpose of the present study is to systematically evaluate the effectiveness of different noise reduction strategies (NRS) using nuisance variables to improve perfusion based FC analysis in two cohorts of healthy adults using state of the art 3D background-suppressed (BS) GRASE pseudo-continuous ASL (pCASL) and dual-echo 2D-EPI pCASL sequences. Five different NRS were performed in healthy volunteers to compare their performance. We then compared seed-based FC analysis using 3D BS GRASE pCASL in a cohort of 12 children with ASD (3f/9m, age 12.8 ± 1.3 years) and 13 typically developing (TD) children (1f/12m; age 13.9 ± 3 years) in conjunction with NRS. Regression of different combinations of nuisance variables affected FC analysis from a seed in the posterior cingulate cortex (PCC) to other areas of the default mode network (DMN) in both BOLD and pCASL data sets. Consistent with existing literature on BOLD-FC, we observed improved spatial specificity after physiological noise reduction and improved long-range connectivity using head movement related regressors. Furthermore, 3D BS GRASE pCASL shows much higher temporal SNR compared to dual-echo 2D-EPI pCASL and similar effects of noise reduction as those observed for BOLD. Seed-based FC analysis using 3D BS GRASE pCASL in children with ASD and TD children showed that noise reduction including physiological and motion related signals as nuisance variables is crucial for identifying altered long-range connectivity from PCC to frontal brain areas associated with ASD. This is the first study that systematically evaluated the effects of different NRS on ASL based FC analysis. 3D BS GRASE pCASL is the preferred ASL sequence for FC analysis due to its superior temporal SNR. Removing physiological noise and motion parameters is critical for detecting altered FC in neurodevelopmental disorders such as ASD.

## Introduction

Functional connectivity (FC) analysis to compute functionally connected networks (FCNs) has become a major imaging approach to investigate the brain's organization and function. Moreover, comparing different cohorts such as elderly subjects to young adults, or healthy control groups to psychiatric populations, have identified patterns of altered connectivity within specific FCNs. In the past few years, however, it has become evident that there are several potential confounding factors that may lead to spurious findings when not properly addressed. Physiological noise such as fluctuations in respiratory and cardiac cycles or head movements can influence BOLD signal intensities in fMRI. This is particularly relevant since different study cohorts could exhibit different patterns or amounts of such confounding factors (e.g., children, elderly, and psychiatric patients tend to have more difficulties to lay motionless inside the MR scanner). Accordingly, using nuisance variables to account for noise related signal fluctuations in BOLD-fMRI based FC analysis has been shown to be imperative to minimize or avoid potential confounding effects of motion or other physiological factors (e.g., respiration and heart rate) on network connectivity measures (Murphy et al., 2013).

Physiological fluctuations or changes in cardiac pulsation and respiratory cycles can cause changes in blood CO_2_ pressure (Wise et al., 2004), which in turn influences the BOLD signal. Hence variability in respiration and cardiac pulsation could give rise to spuriously correlated signals in distributed brain areas (Birn, 2012). Furthermore, the set of brain areas affected by these physiological fluctuations could resemble the patterns associated with certain FCNs (Birn et al., 2008). Accordingly, separating physiological noise from BOLD signal fluctuations increases sensitivity for detecting neuronal related FCNs. While these variations in physiological parameters are ideally measured by concurrent recordings with pulse oximetry and respiration belt (Chang and Glover, 2009b), data driven techniques have been proposed to estimate nuisance regressors from the fMRI data itself. Furthermore, regions that are unlikely to exhibit neuronal related BOLD signal changes such as in cerebro-spinal fluid (CSF) or white matter (WM) have been used to efficiently remove these physiological variations (Birn et al., 2008; Weissenbacher et al., 2009).

In addition to physiological noise, recent observations indicate also that head movements during the MR acquisition can have detrimental effects on FC measures (Power et al., 2012; Satterthwaite et al., 2012; Van Dijk et al., 2012). Head motion in the magnetic field perturbs the spin history and can introduce spurious signal variances that tend to be more similar locally than between distant brain areas. This biases FC analyses toward increased local correlations and reduced long-range correlations, a critical issue when comparing subject cohorts that might differ in their ability to lie still (e.g., children or psychiatric patients). Indeed children and psychiatric cohorts have displayed this pattern of increased local but reduced long-range FC, raising the critical question as to whether and to what extent these findings reflect motion effects. A variety of approaches to deal with motion effects in FC analyses have since been proposed, most of which include nuisance variables to regress out potential signal fluctuations related to head movements by using motion parameters estimated from rigid body volume alignments (for review see Power et al., 2015). In summary, in BOLD fcMRI several confounding factors have been identified and strategies have been proposed to minimize their influences.

Besides BOLD fcMRI, Arterial Spin Labeling (ASL) datasets have been recently used to compute FCNs (Viviani et al., 2011; Liang et al., 2012; Jann et al., 2015a) (review Chen et al., 2015). This approach was made feasible by technical advances in state-of-the-art ASL pulse sequences resulting in improved signal-to-noise ratio (SNR) and temporal stability (Chen et al., 2011; Vidorreta et al., 2013). These technical advances include pseudo-continuous ASL (pCASL) (Wu et al., 2007; Dai et al., 2008), background suppression and three-dimensional (3D) fast imaging sequences such as GRASE (a hybrid of gradient and spin echo) or stack of spirals. In addition to improved acquisition techniques, physiological noise regression in ASL has been shown to increase temporal SNR (Wang, 2012). To date, however, no study has systematically investigated the effect of noise reduction, using the same nuisance variables as proposed for BOLD, on ASL based FC. Therefore, the primary purpose of this study was to investigate the effect of motion and physiological noise reduction on ASL based FC. A second goal of this study was to apply the optimal noise-reduction strategy for ASL based FC analysis in a cohort of children with autism spectrum disorders (ASD) and typically developing children.

## Methods

All adult neurotypical participants in this study gave written informed consent according to a research protocol approved by the UCLA Institutional Review Board. Inclusion criteria of healthy volunteers included no history of psychiatric or neurological disorders, and no contraindications to MRI scan. Scans were performed on a 3T Siemens TIM Trio scanner, using body coil as the transmitter and 12-ch head coil as the receiver. We acquired ASL and BOLD data in 10 healthy young participants (6f/4m; age [mean ± sd] = 22 ± 3 years) with a 3D background-suppressed (BS: 85% suppression) GRASE pCASL sequence (60 label/control pairs, TR/TE/τ/PLD = 4000/22/1200/1000 ms; 26 slices, 64 × 64 matrix, voxel-size 3.44 × 3.44 × 5 mm^3^) and a standard 2D EPI BOLD sequence (240 Volumes, TR/TE = 2000/30 ms, 30 slices, 64 × 64 matrix, slice thickness = 4 mm with 1 mm gap). For comparison, a separate cohort of 10 healthy volunteers (7f/3m; age [mean ± sd] = 25.7 ± 8 years) underwent resting state fMRI scans using a dual-echo 2D EPI pCASL sequence (128 label/control pairs, TR/TE1/TE2/τ/PLD = 4000/10/25/1200/1500 ms; 18 slices, 64 × 64 matrix, voxel-size 3.44 × 3.44 × 6 mm^3^) to simultaneously acquire ASL and BOLD data. All datasets were first realigned to account for spatial motion displacements (for ASL separately for label and control images). Five different regression models using different sets of nuisance variables [here termed Noise Reduction Strategies (NRS)] were then performed:

- *NRS1*: no nuisance variables for noise reduction.
- *NRS2*: 6 motion parameters (3 translations x, y, z and 3 rotations α, β, γ) and their 1st derivatives.
- *NRS3*: same as NRS2 plus additional regressor for Framewise Displacement (FD). FD was computed following the procedure described by Power et al. (2012). Rotational displacements were recomputed to millimeters of displacement on a sphere with 5 cm radius. The volume by volume (framewise) head displacement in translational and recomputed rotational parameters were then calculated and summed up. Mean FD (±SD) in mm for the groups in each dataset were: 3D GRASE pCASL 0.244 (±0.065), standard BOLD 0.192 (±0.060), 2D dual-echo pCASL 0.179 (±0.066) and dual-echo BOLD 0.153 (±0.049). *T*-tests did not reveal a significant difference between ASL and BOLD within the groups (t_3D_ = 1.81; *p* = 0.086/t_2D_ = 0.996; *p* = 0.333), nor between groups for BOLD (*t* = 1.612; *p* = 0.124). There was a small difference showing slightly higher motion in 3D pCASL than 2D pCASL (*t* = −2.194; *p* = 0.042).
- *NRS4*: White matter and CSF fluctuations (mean signal fluctuations within brain segmentation tissue probability masks thresholded at 0.95 for WM and 0.85 for CSF and coregistered/resampled to functional images).
- *NRS5*: NRS3 + NRS4.

CBF images were computed for all NRSs (one compartment model, pair-wise subtraction of Label/Control images) (Alsop et al., 2015). BOLD and CBF images were coregistered to individual anatomical scans, normalized to MNI template and smoothed with an 8 mm FWHM Gaussian kernel.

### Connectivity analysis

FC analysis was performed with Seed Based Correlation Analysis (SBA) using the posterior cingulate cortex (PCC) as a seed (template seed from Shirer et al., 2012) to identify the Default Mode Network (DMN). DMN maps for each NRS in all four datasets as well as overall DMNs for the four datasets were calculated by one-sample *t*-tests to identify all areas with correlations significantly greater than zero across all subjects. We determined the similarity of the DMN maps derived using different NRSs between each other as well as to a template BOLD-DMN (Shirer et al., 2012) and template ASL-DMN (Jann et al., 2015a) using Dice Similarity Coefficients (Dice, 1945; Jann et al., 2015a), which compares the number of common voxels between two maps based on the formula DSC (A,B) = 2(A∩B)/(A + B), where A and B are the two maps.

To investigate the effect of NRS on the often-discussed long-range connectivity between PCC and anterior cingulate/medial prefrontal cortex (ACC/mPFC) (Power et al., 2012, 2015; Satterthwaite et al., 2012; Van Dijk et al., 2012), we calculated the correlation between those two ROIs based on the template DMN nodes (Shirer et al., 2012) using different NRSs. FC changes due to different NRSs were further investigated by a voxel-wise analysis on the individual subjects' SBA connectivity maps for each NRS by computing voxel-wise repeated-measures ANOVA and *post-hoc* ROI based paired *t*-tests.

### Distance related effects of motion

We further investigated the relationship between spatial distance, the use of head motion related nuisance variables and FC changes in BOLD and pCASL data, respectively. Specifically, we parcellated the brain into 264 spherical ROIs defined by the Power-Atlas (Power et al., 2012). For the parcellated data we then computed the cross-correlation matrix using data processed with NRS4 (WM/CSF regression only) and NRS5 (WM/CSF regression + motion regression), respectively. Subtraction of the two cross-correlation matrices provides the difference in connectivity between any two ROIs (ΔFC) between NRS5 and NRS4. Plotting these ΔFC values against the Euclidean distance between the respective ROIs and fitting a linear equation to these plots examined the presence of a relation between ΔFC and spatial distance (Power et al., 2014, 2015).

### Effects on temporal SNR and global-CBF quantification

Finally, we estimated tSNR within gray matter and performed an ANOVA on these values to test for significant improvements in tSNR following noise reduction. Global mean CBF was also compared to test whether NRS affects mean CBF quantification between the two pCASL sequences.

### Application of NRS in children with ASD

To investigate the effects of NRS on seed-based FC analysis in a clinical cohort, we compared FC differences between a group of 12 children with ASD (3f/9m, age 12.8 ± 1.3 years; IQ = 107.0 ± 14.9) and an age and IQ matched group of 13 typically developing (TD) children (1f/12m; age 13.9 ± 3 years; IQ = 104.8 ± 14.4). Subjects and parents provided written consent according to the guidelines specified by the UCLA Institutional Review Board. Clinical diagnosis of ASD was confirmed with the Autism Diagnostic Observation Schedule (ADOS; Lord et al., 2000), Autism Diagnostic Interview-Revised (ADI-R; Lord et al., 1994) and best clinical judgment. Mean ADOS severity score was 7.6 (range 6–10). Statistical tests to compare group characteristics were not significant: Mann-Whitney *U*-Test for age (*U* = 44, *z* = 1.822, *p* = 0.068) and IQ (*U* = 73.5, *z* = −0.2178, *p* = 0.826) and Chi-square test with Yates correction for small samples for gender (0.401, *p* = 0.527).

We used the 3D BS GRASE pCASL sequence to acquire CBF data in these two groups given its favorable temporal characteristics shown in the above analyses. Preprocessing was identical as described above and NRS4 and NRS5 were compared to NRS1 in these cohorts. FC from the PCC-seed was computed for each subject and NRS. Within group analyses included comparisons between NRSs using voxel-wise paired-sample *t*-tests. In addition, between-group comparisons were performed by voxel-wise two-sample *t*-tests (correction for multiple comparison at α < 0.05 was done by cluster-size estimation, CSE for NRS0 = 133 voxels, NRS4 = 115 voxels and NRS5 = 114 voxels). This analysis will reveal the effects of different NRSs on the outcome of ASL-FC differences between ASD and TD. To minimize the effects of differences in head motion between the groups, the ASD and TD groups were also matched for the amount of motion: mean frame-wise displacement (FD) for ASD was 0.453 ± 0.238 and TD 0.392 ± 0.241 (*t*-test *t* = −0.641, *p* = 0.528).

## Results

**Figure 1:**
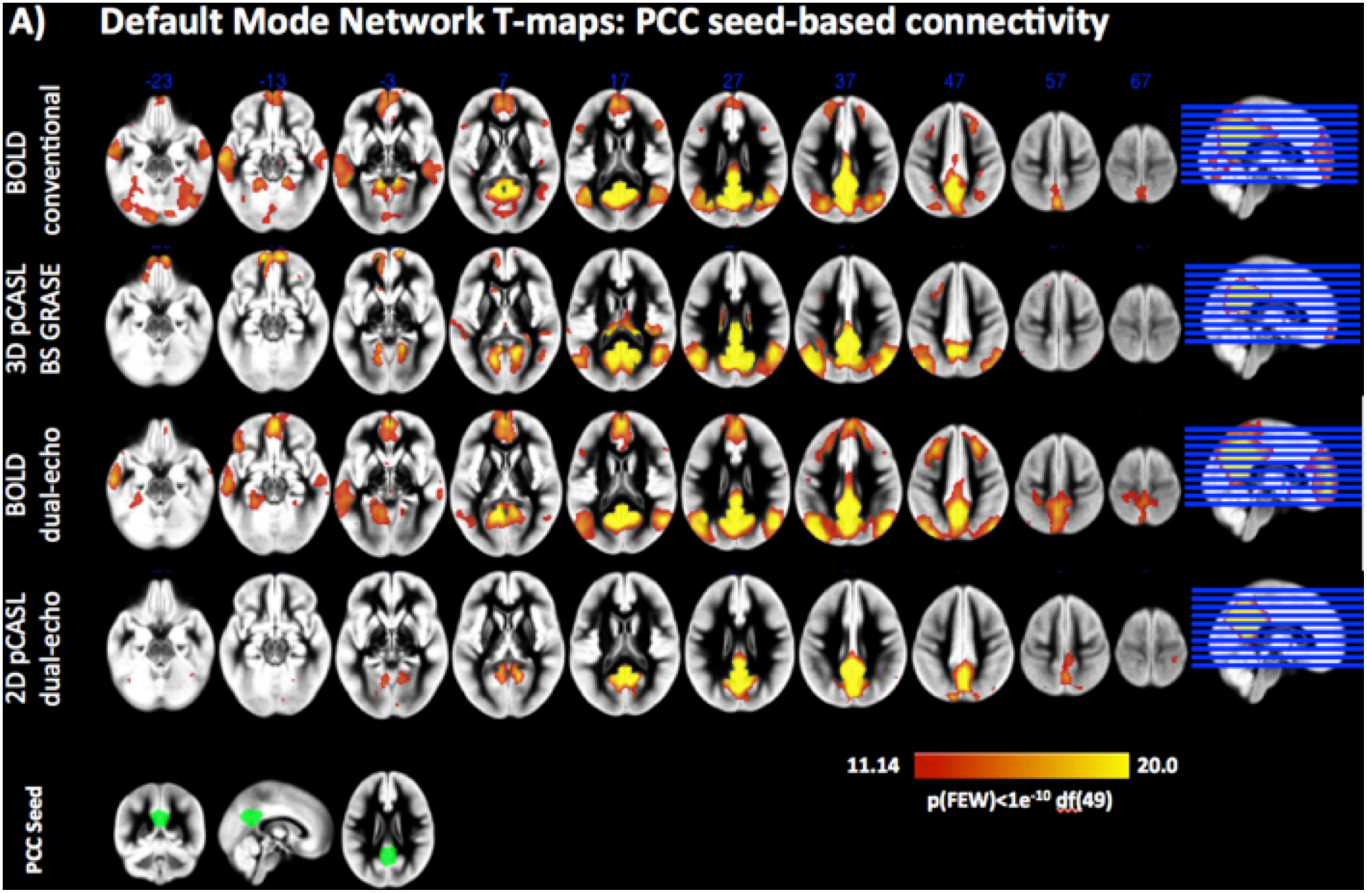
**Statistical t-maps displaying the spatial pattern of the DMNs as identified by Seed based Correlation analysis from a PCC seed (Lower left, green) on pCASL and BOLD datasets**.

**Figure 2:**
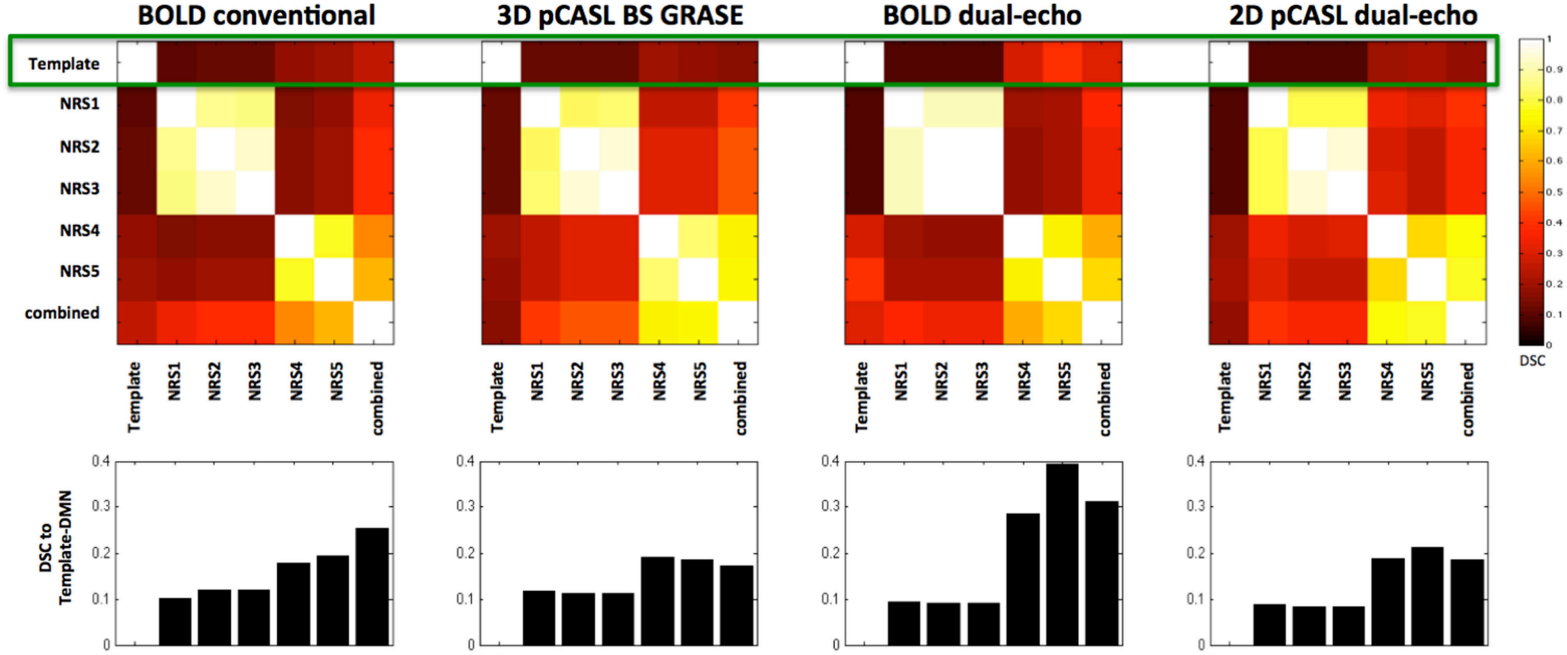
**Dice Similarity Coefficients (DSCs) between the spatial maps of the DMNs after different stages of noise reduction (NRS1–5)**. Template DMN derived from Shirer et al. (2012). Combined DMN computed as one-sample *t*-test across spatial correlation maps of all subjects and NRS. Clusters along the diagonal indicate that WM/CSF regression has large effect on similarity. Specifically after WM/CSF correction (NRS4/5) DSC values to the template DMN are increased (green rectangle and also bar-plots below cluster-plots), indicating increased spatial specificity of the DMN.

Both pCASL and BOLD data showed correlation maps using the PCC as the seed that resemble the DMN. Figure 1 displays the PCC-Seed and the DMNs computed as t-maps across all NRSs for each dataset thresholded at family wise corrected *p* < 1e^−10^. Dice Similarity Coefficients (DSCs) to the template BOLD-DMN (Shirer et al., 2012) and the template ASL-DMN (Jann et al., 2015a), respectively, are listed in Table 1. NRS1–3 showed low similarity while NRS4&5 showed greater overlap with the DMN templates. Furthermore, 2D pCASL showed the lowest DSC values, especially to the template ASL-DMN. Figure 2 displays the cross-comparison of NRSs within each dataset to the template BOLD-DMN. Notably, DSC values between the separate NRS-DMNs showed that the DMN-maps without WM/CSF correction (NRS1–3) were highly similar to each other, while the DMNs with WM/CSF correction (NRS4, 5) showed high similarity to each other.

**Table 1:** **Dice Similarity Coefficients (DSCs) for all NRS in each condition to a template BOLD-DMN (Shirer et al., 2012) and a template ASL-DMN (Jann et al., 2015a), respectively**.

	**NRS1**	**NRS2**	**NRS3**	**NRS4**	**NRS5**	**Combined**
**TEMPLATE BOLD-DMN**						
BOLD conventional	0.10	0.12	0.12	0.18	0.19	0.25
3D BS GRASE pCASL	0.12	0.11	0.11	0.19	0.19	0.17
BOLD dual-echo	0.09	0.09	0.09	0.28	0.39	0.39
2D pCASL	0.09	0.08	0.08	0.19	0.21	0.19
**TEMPLATE ASL-DMN**						
BOLD conventional	0.24	0.27	0.27	0.40	0.43	0.51
3D BS GRASE pCASL	0.36	0.40	0.40	0.51	0.49	0.55
BOLD dual-echo	0.24	0.23	0.23	0.51	0.55	0.58
2D pCASL	0.29	0.26	0.26	0.33	0.32	0.35

The column termed combined represents the DMN t-maps across all subjects and NRSs as displayed in Figure 1.

### Effects of NRS on FC

**Figure 3:**
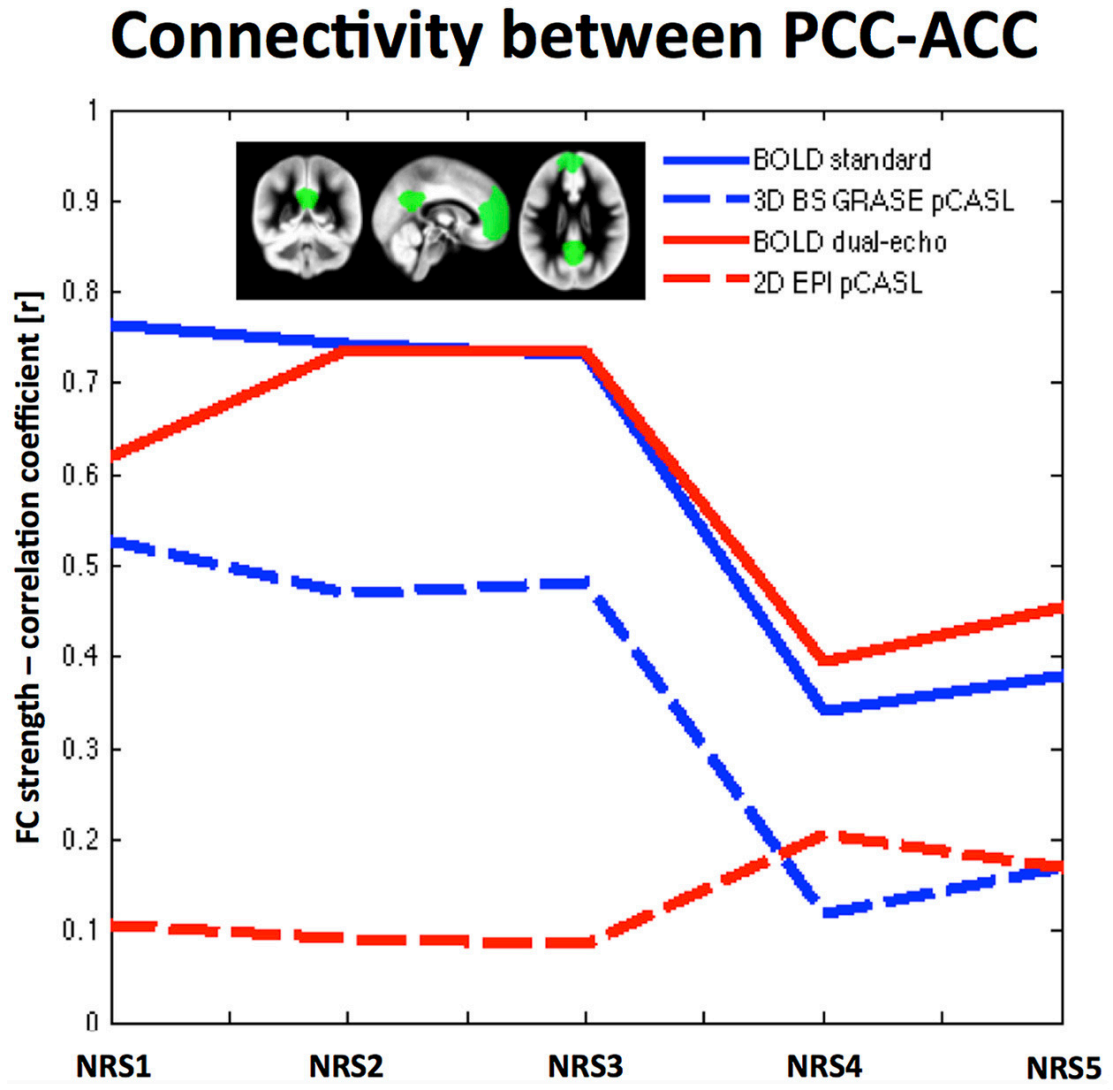
**Region-to-Region correlation between two template areas of the DMN: the PCC and the ACC/mPFC (shown in green in the small inlet figure)**. Overall BOLD exhibits stronger correlation values than ASL. Noise reduction shows similar behavior in FC changes for both BOLD datasets (solid lines) as well as the 3D BS GRASE pCASL data (blue dashed line), while showing little effect for 2D pCASL. A more regionally detailed analysis of these effects is displayed in Figure 4.

Analysis of the connectivity between PCC and ACC/mPFC ROIs in both BOLD datasets showed a general decrease of FC after WM/CSF regression [F_BOLDconv(4, 9)_ = 25.53, *p* < 1e-10, t_NRS0vsNRS4_ = 5.34, *p* = 0.0005; F_BOLDdual−echo(4, 9)_ = 12.49, *p* < 1e^−10^, t_NRS0vsNRS4_ = 2.69, *p* = 0.025], while motion parameter regression showed a tendency to slightly increase FC (*t*-test between NRS5 and NRS4 = t_BOLDconv_ = 1.55, *p* = 0.155; t_BOLDde_ = 1.14, *p* = 0.284). The same trend was observed for 3D GRASE pCASL [F_3DpCASL(4, 9)_ = 75.5, *p* < 1e^−10^; t_NRS0vsNRS4_ = 14.19, *p* < 0.0001 and *t*-test NRS5 vs. NRS4: t_3DpCASL_ = 1.51, *p* = 0.165]; however, FC values were overall lower. For 2D pCASL there was little to no correlation between the two ROIs and no significant effect in the ANOVA (F_2DpCASL_ = 1.4, *p* = 0.254). Mean values of correlation coefficient across subjects for all NRSs and datasets are displayed in Figure 3.

**Figure 4:**
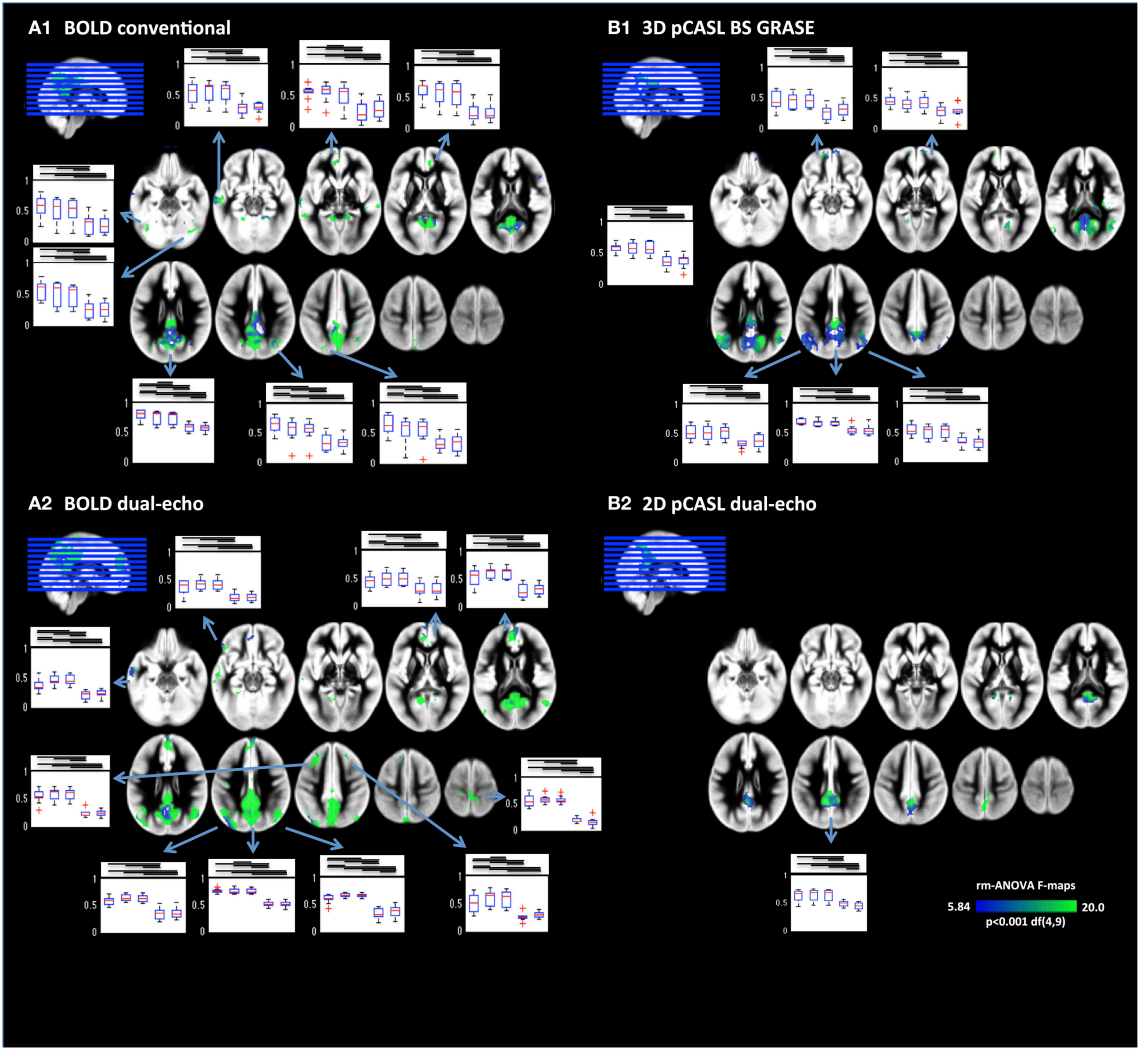
**Voxel-wise repeated-measures ANOVA F-maps highlighting the areas with significant FC changes after NRS**. Results are limited to areas within the DMNs as displayed in Figure 1. Box plots display the regional FC values for NRS1–5. Generally physiological noise reduction (WM and CSF fluctuations) reduced FC significantly (bars above box plots indicate significance between NRS-FC values). Motion correction had more subtle effects on FC but nevertheless significant increases can be seen in several areas mainly in the frontal cortex.

A more detailed analysis of NRS effects throughout the DMN was performed by voxel-wise repeated-measures ANOVA (Figure 4). Results revealed that FC between PCC and mPFC/ACC were modified by NRS. Details for all ROIs including the results of the repeated-measures ANOVAs are listed in Table 2. For every ROI showing an effect of NRS, the boxplots represent the FC values (median and 75% interval across subjects) after different NRSs, revealing the directions of FC changes (i.e., increases or decreases). Moreover, the horizontal lines above the boxplots indicate the significance of *post-hoc* paired *t*-tests (*p* < 0.05) between any NRSs (*t* and *p* values for all *post-hoc t*-tests can be found in Supplemental Table 1). Similar to the analysis of connectivity between the PCC and ACC/mPFC ROIs, the voxel-wise ANOVA and *post-hoc t*-tests indicated that WM/CSF signal regression significantly reduces FC throughout the DMN. Furthermore, using head movement related nuisance variables in addition to WM/CSF (comparison between NRS5 and NRS4) tended to increase long-range FC from PCC to frontal areas while reducing local (within PCC) FC (Table 2). This distance-related effect was further investigated in a highly parcellated seed based approach.

**Table 2:** **Clusters showing a change in functional connectivity across noise reduction strategies (NRS)**.

	**Cluster size**	**Peak MNI coordinate**			**peak F**	**Anatomical area**	**BA**	**rm-ANOVA**		**Effects of motion regression**	
**Cluster**	**#voxels**	***x***	***y***	***z***				***F*_(4, 9)_**	***P***	***Post-hoc t*-test NRS5-NRS4**	***p***
**3D BOLD**											
1	91	34.0	−70.1	−23.9	47.3898	Fusiform_R	19	6.02	8.10E-04	−0.40	0.6996
2	329	−52.0	−14.1	−9.9	61.1242	Temporal_Mid_L	21	4.45	0.005	−0.01	0.9912
3	5290	0.0	−60.1	56.1	97.9045	Precuneus/posterior cingulate	(7/31)	15.95	1.34E-07	−1.20	0.2598
4	54	−18.0	−44.1	−11.9	38.5708	Fusiform_L	19	13.77	6.60E-07	−1.22	0.2534
5	53	−6.0	41.9	2.1	29.5438	Cingulum_Ant_L	32/10	4.03	0.0084	2.42	0.0386^*^
6	52	10.0	43.9	8.1	46.4137	Cingulum_Ant_R	32	11.14	5.56E-06	0.08	0.9390
7	259	28.0	−76.1	40.1	28.0085	Occipital_Sup_R	19	7.67	1.41E-04	−0.59	0.5716
8	86	−20.0	−62.1	38.1	42.1778	Parietal_Sup_L	7	4.37	0.0055	−0.72	0.4925
**3D ASL**											
1	129	14.0	63.9	6.1	23.8028	Frontal_Sup_Medial_R	(11/10)	6.67	4.03E-04	0.54	0.6032
2	184	−10.0	45.9	−3.9	44.5863	Cingulum_Ant_L	32	6.29	6.03E-04	3.34	0.0087^*^
3	4003	4.0	−44.1	46.1	37.23	Precuneus/posterior cingulate	7,31	3.17	0.0248	−0.61	0.5562
4	864	50.0	−46.1	28.1	28.0536	SupraMarginal_R	39	11.67	3.55E-06	0.11	0.9122
5	70	28.0	−34.1	12.1	33.0022	sub lobar		4.31	0.0059	−0.05	0.9604
6	1338	−56.0	−54.1	28.1	27.4496	SupraMarginal_L	39	6.06	7.78E-04	2.00	0.0770
**2D BOLD**											
1	333	−60.0	−24.1	−5.9	40.2981	Temporal_Mid_L	21	0.86	0.499	1.35	0.2113
2	81	−46.0	25.9	−9.9	50.8921	Frontal_Inf_Orb_L	47	4.32	0.0059	0.13	0.8987
3	91	−6.0	43.9	−3.9	19.2798	Cingulum_Ant_L	10.11	2.27	0.0805	0.59	0.5717
4	5609	4.0	−28.1	44.1	243.5297	Cingulum_Mid_R	31	0.85	0.5048	−1.46	0.1791
5	975	8.0	31.9	24.1	85.5258	Cingulum_Ant_R	10,32,9	2.74	0.0435	1.56	0.1521
6	833	48.0	−56.1	40.1	97.0885	Angular_R	39	2.97	0.0321	2.64	0.0268^*^
7	1014	−40.0	−54.1	34.1	66.9547	Angular_L	19,39	2.91	0.0347	0.99	0.3491
8	314	−22.0	15.9	44.1	251.0319	Frontal_Mid_L	8	4.41	0.0053	−0.15	0.8875
9	74	26.0	23.9	52.1	55.824	Frontal_Sup_R	8	3.12	0.0265	2.03	0.0735
10	188	8.0	−44.1	64.1	154.553	Paracentral_Lobule_R	6	0.49	0.745	−2.46	0.0363^*^
**2D ASL**											
1	2273	4.0	−36.1	58.1	72.8902	Posterior Cingulate	31,7	6.14	7.11E-04	−3.26	0.0099^*^

Listed are cluster coordinates, size, and anatomical location as well as statistical test results for repeated measures ANOVA and post-hoc T-test between NRS5 vs. NRS4 (FC changes due to motion regression).

* Indicate significant differences between NRS5 vs NRS4.

### Distance dependence of motion-regression effects

**Figure 5:**
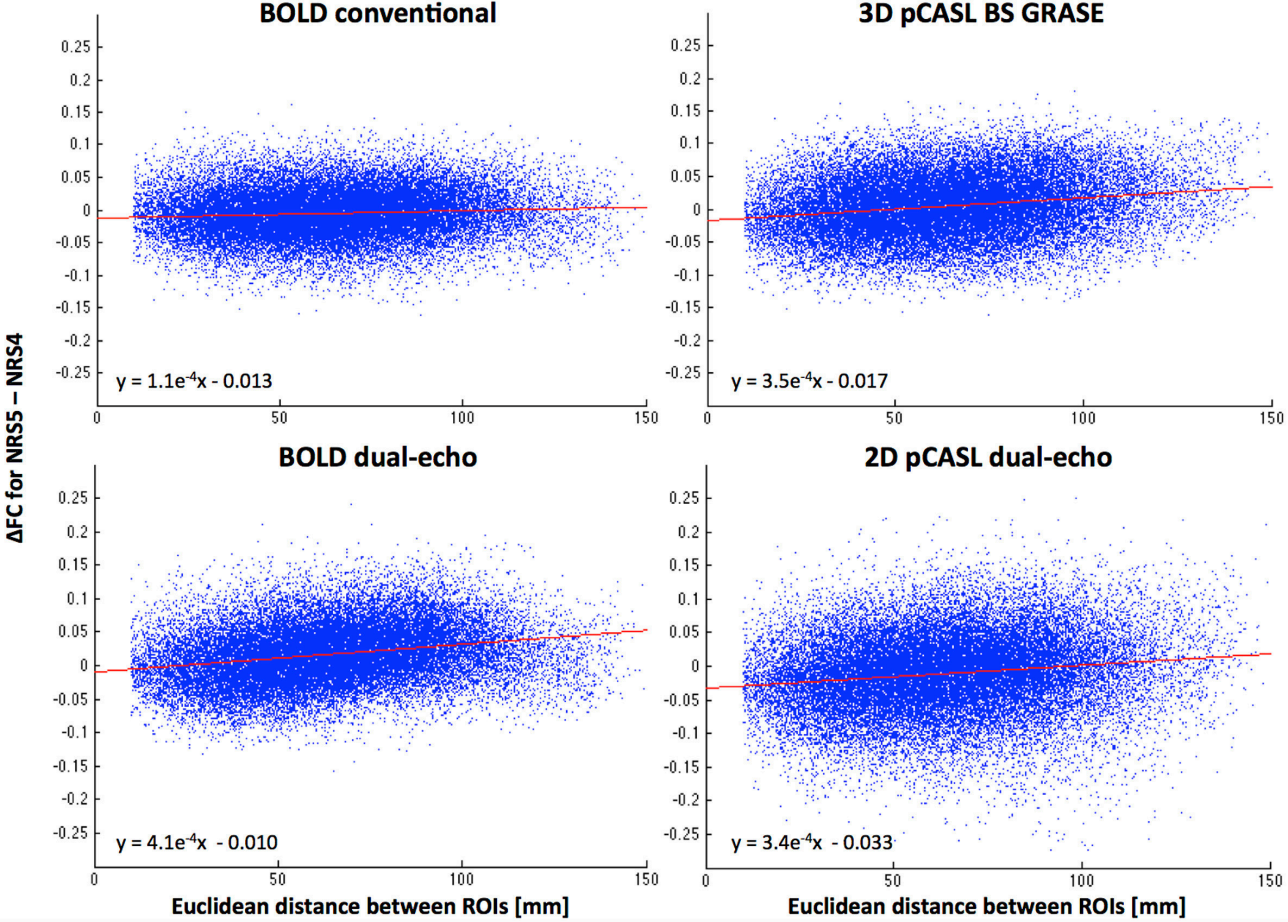
**Distance dependence of FC changes between NRS4 and NRS5 (indicating motion correction effects only, after correction for physiological noise)**. Scatter plots reveal that FC between more distant areas is increased more than for proximal areas. Red lines represent the linear fit between distance and ΔFC (see also equations).

The scatter plots in Figure 5 suggest a relationship between motion correction effects (after WM/CSF signal regression) and the distance between the connected ROIs. Specifically, ΔFC was increasingly positive the farther apart any two areas, thus yielding increased long-range connectivity and reduced or constant local short-range connectivity.

### NRS effects on temporal SNR and global CBF

**Figure 6:**
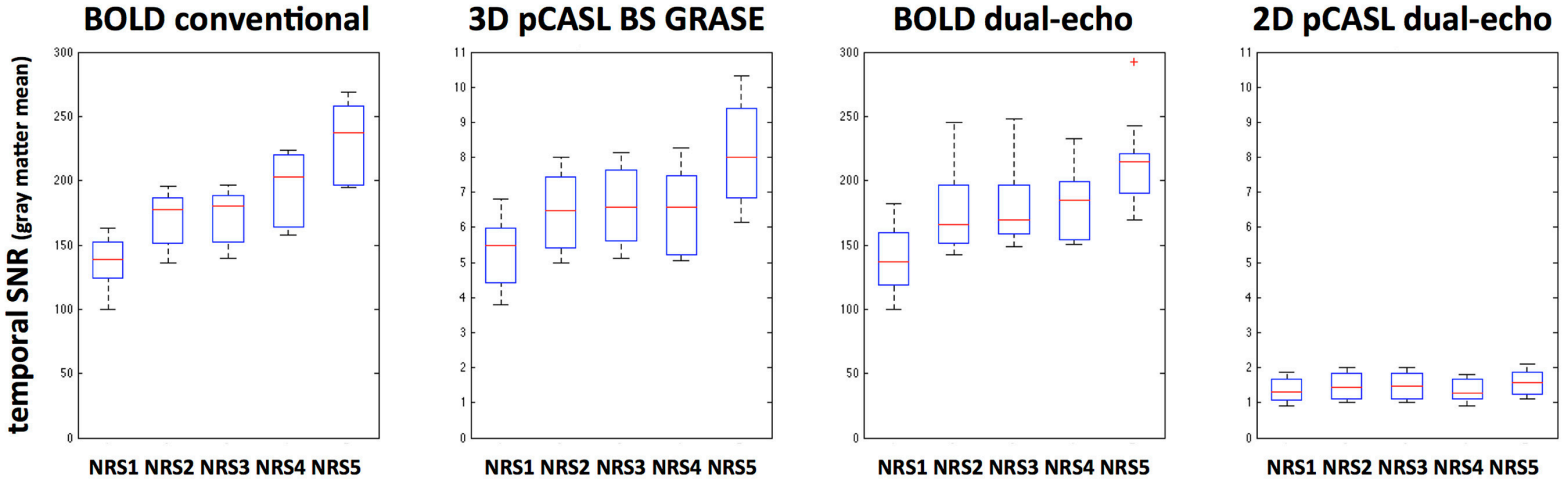
**Boxplots indicating tSNR increases after different noise reduction strategies (NRS1–5) in all analyzed MRI sequences**. Significant effects were found for all but the 2D dual-echo pCASL datasets.

Global tSNR in BOLD was expectedly higher than that of pCASL; furthermore, 3D pCASL showed 4.4 ± 0.4 times higher global tSNR than 2D pCASL. Differences in tSNR after NRS were observed for all modalities and with a similar behavior suggesting that motion regression increases tSNR and combining motion regression with WM/CSF regression results in highest increase in tSNR (Figure 6). This observation is supported by repeated-measures ANOVA analyses for both BOLD and 3D GRASE pCASL sequences: *F*_(4, 9)*BOLD*−*S*_ = 7.5, *p* < 0.001, F_BOLD−DE_ = 21.92, *p* < 5e^−10^, F_ASL−3D−BS_ = 7.78, *p* < 7.5e^−5^, whereas there was no significant effect for 2D dual-echo pCASL: F_ASL−2D−DE_ = 0.73, *p* = 0.58.

Along with the increase in temporal SNR for 3D GRASE pCASL, global mean CBF was also slightly higher for NRS4&5 than those observed for NRS1–3 [*F*_(4, 9)_ = 63.36, *p* < 0.00001]. An opposite effect was observed for 2D dual-echo pCASL where regression of WM/CSF signals (NRS4&5) slightly reduced global mean CBF [*F*_(4, 9)_ = 3.36, *p* < 0.02], as shown in Table 3.

**Table 3:** **Mean global CBF for both pCASL sequences and all NRSs**.

**Global CBF [ml/100g/min]**	**NRS1**	**NRS2**	**NRS3**	**NRS4**	**NRS5**
2D EPI pCASL	59.66 ± 11.45	59.79 ± 11.45	59.79 ± 11.45	58.21 ± 9.77	58.17 ± 9.88
3D BS GRASE pCASL	59.99 ± 9.70	59.98 ± 9.68	59.99 ± 9.69	64.37 ± 10.78	64.37 ± 10.78

No differences were observed.

### Results of NRS in children with ASD vs. TD children

#### Within-group

Comparing correlation maps seeded from the PCC revealed differences in long-range connections to the frontal cortex after noise reduction in both groups (TD and ASD children). Specifically, differences between NRS4 and NRS5 revealed increased long-range and decreased local correlations within the DMN, which are in accordance with the general observations of motion-regression effects (Supplemental Figure 1). In TD children, we found increased correlation to superior frontal gyri and to the hippocampi, as well as reduced local connectivity in PCC. In ASD children, we observed increased correlations with the orbitofrontal cortex (OFC) and similarly reductions in FC in PCC. Furthermore, we observed increases in anti-correlation to areas associated to other large-scale networks in autism: from PCC to the dorsal ACC, part of salience network, as well as regions of the motor network. Hence, in addition to within network effects (DMN), noise reduction might also increase the separation between networks.

#### Between-group

**Figure 7:**
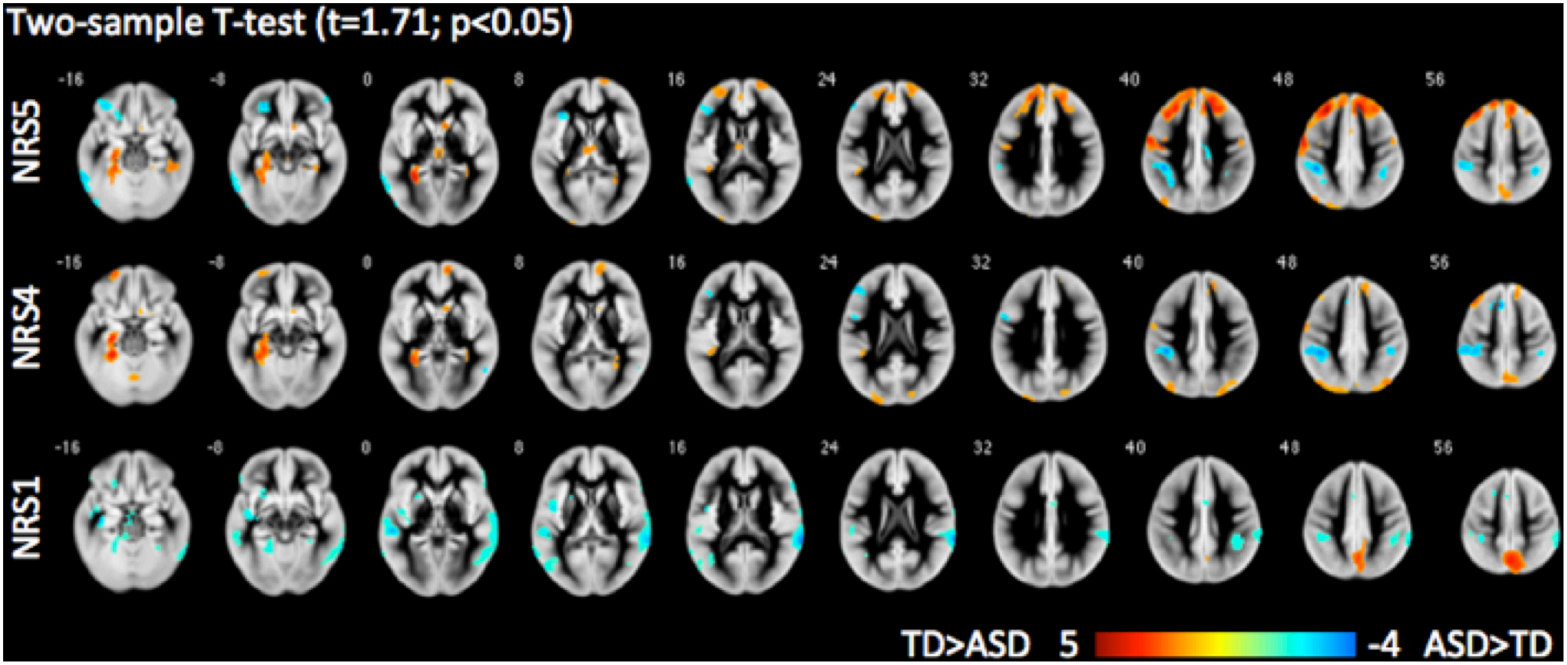
**Group differences between typically developing (TD) children and children with autism spectrum disorder (ASD) for different NRS in 3D BS GRASE pCASL data: NRS1 no noise reduction, NRS4 WM/CSF fluctuations removed, NRS5 WM/CSF and motion regression**. While seed based FC (from PCC) in NRS1 did not reveal differences in frontal areas, NRS5 clearly delineates reduced long-range connections in ASD compared to TD.

Direct comparisons between the TD and ASD groups revealed evidence highlighting the importance of noise reduction (Figure 7). While group differences without any noise-reduction (NRS1) showed decreased local FC in the precuneus and increased FC to lateral temporal areas bilaterally in the ASD group as compared to the TD group, group differences after noise regression (NRS4&5) revealed areas with reduced long-range FC from PCC to the dorsal portion of the prefrontal cortex and parahippocampal gyri in the ASD vs. the TD group. The areas showing reduced connectivity with the lateral temporal lobes in the ASD group were no longer evident.

## Discussion

FC analysis has become a major tool to assess the functional organization of brain networks as well as their integrity or alterations in clinical populations. However, to be clinically applicable, possible confounding factors for FC analysis need to be identified, understood and accounted for. For BOLD based FC such effects include physiological noise related to pulsatile fluctuations of the blood flow caused by heart beat (Shmueli et al., 2007; Chang et al., 2009) as well as changes in BOLD signal due to variations in rate and depth of respiration (Birn et al., 2006; Birn, 2012). More recently, it has been shown that even slight head movements can affect FC analysis outcomes (Power et al., 2012; Van Dijk et al., 2012).

By using different sets of nuisance variables (here termed Noise Reduction Strategies: NRS) representing noise from physiological noise sources and head movements in two separate BOLD and pCASL implementations, our study showed that accounting for physiological noise and motion-induced effects could indeed alter the connectivity strength and hence spatial maps of the DMN. For BOLD rs-fMRI, these effects have been described and were replicated in our study. For ASL, so far little has been known regarding how noise reduction affects ASL signal and ASL based FC analysis.

### Noise reduction effects in bold based FC

Our findings generally align well with what has been described with regard to noise reduction effects in BOLD based FC analysis. First, regression of physiological noise related nuisance variables from WM and CSF signal fluctuations (Dagli et al., 1999; Windischberger et al., 2002; Birn et al., 2008; Weissenbacher et al., 2009; Jo et al., 2010) reduces connectivity in several brain areas but at the same time increases the spatial specificity of FC maps (Chang and Glover, 2009a; Birn, 2012; Power et al., 2015). The correlation maps generated by NRS4 and NRS5 are highly restricted to areas of the DMN while NRS1–3 display more widespread correlation maps that comprise areas affected by respiratory and heart rate pulsatility (Birn et al., 2008). Statistical comparisons using DCSs further demonstrated that the spatial maps for NRSs including physiological noise regression improved their similarity to the template DMN. A decrease in connectivity strength after removing physiological fluctuations is expected since noise induced spurious correlations are removed from the signal (Weissenbacher et al., 2009). Furthermore, temporal SNR was improved by noise reduction indicating reduced signal variance.

Using head motion nuisance regressors showed less pronounced effects. For conventional BOLD and dual-echo BOLD, frontal regions showed a trend of FC increases between NRS4 and NRS5 as well as FC decreases in the medial posterior cortex and PCC. Significant increases were only observed in ACC for the conventional BOLD dataset and the right angular gyrus for dual-echo BOLD (Table 2). However, the data overall suggest that reduction of head movement related signals improves FC strength between anterior and posterior areas. This effect has attracted wide interest in recent years since the head movement effects are subtle and can cause group differences between cohorts with different movement profiles (e.g., patient populations or children Van Dijk et al., 2010; Satterthwaite et al., 2012). Notably, in this study, there were no head movement differences within the neurotypical adult groups nor between the ASD and TD groups as evidenced by mean framewise displacement. Moreover, the spatial extent of DMN and regional effects of NRS onto FC within the DMN were compared within datasets separately (except for the between-group comparison of ASD vs. TD discussed below). Our participants further showed only small amount of motion hence changes were expected to be subtle. In a further analysis, we segregated the cortex into 264 regions and computed the distance dependence of FC due to reduction of head motion effects. This confirmed that long-range connections show proportionally larger increase in FC than connections between more proximal areas. This finding is in line with prior evidence that motion affects long- and short-range connections differently (Power et al., 2012, 2014; Van Dijk et al., 2012). Comparing our linear fitting results to recent work by Power et al. (2012, 2015) confirms that the distance dependency effects are in the same order of magnitude.

In summary, in both BOLD datasets the observed effects of noise reduction are in agreement with previous work, highlighting the importance of taking into account the effect of physiological and motion related confounds in FC analyses.

### Noise reduction effects in ASL based FC

ASL based FC has recently gained interest in the research community (Chuang et al., 2008; Zou et al., 2009; Viviani et al., 2011; Jann et al., 2013; Dai et al., 2015; Jann et al., 2015a, for recent review see Chen et al., 2015) and in clinical studies (Orosz et al., 2012; Kindler et al., 2013; Jann et al., 2015b), since it provides not only assessments of functional brain networks but also a surrogate measure of metabolism, cerebral blood flow (CBF). Moreover, there appears to be a relation between connectivity strength and local CBF suggesting that increased connectivity of a region is more energy demanding (Liang et al., 2013; Tomasi et al., 2013; Jann et al., 2015a). While the feasibility of ASL based FC and the similarity of the identified networks to BOLD networks has been previously demonstrated (Chuang et al., 2008; Zou et al., 2009; Viviani et al., 2011; Jann et al., 2013, 2015a; Dai et al., 2015), it remains unknown how noise regression in ASL could benefit these analyses. Our results show that 3D GRASE pCASL with background suppression (BS) benefits from noise reduction as temporal SNR (tSNR) significantly increases in a similar manner as for BOLD (Figure 6). For 2D pCASL without BS there was a minor gain in tSNR (Wang et al., 2008; Wang, 2012) although this did not reach significance. Furthermore, we observed that 3D BS GRASE pCASL offers a four-times higher tSNR than that of 2D pCASL. This higher tSNR across all NRSs can mainly be attributed to the background suppression (brain tissue signal suppressed by 85%), while the 3D readout mainly contributes to improved spatial SNR (Vidorreta et al., 2013; Chen et al., 2015; Wang et al., 2015). Based on tSNR measurements, 3D BS GRASE pCASL should be more suitable for CBF based FC analyses than 2D pCASL without BS.

The FC analysis on the CBF datasets demonstrated that the DMN can be detected in both pCASL implementations, albeit with less statistical power in the 2D pCASL due to lower tSNR and/or small sample size of this study. Removing WM and CSF fluctuations to minimize cardiac and respiration related noise prior to FC analysis resulted in reduced FC between PCC and ACC in the ROI based analysis for 3D BS GRASE pCASL whereas no significant effect was found for 2D pCASL. Notably, at the selected statistical threshold, 2D dual-echo pCASL did not show significant correlations between CBF signals in the seed area in the PCC and the anterior part of the DMN (i.e., the mPFC/ACC). It remains to be determined whether using larger samples or lower statistical thresholds will make 2D ASL based FC analysis feasible. Furthermore, as discussed above, the BOLD images acquired at the second echo of the dual echo ASL sequence used in our study showed highly similar network maps, correlation strength and behavior to NRS as the conventional BOLD sequence. On the other hand, the FC strength decrease between PCC and the frontal ROI in 3D BS GRASE pCASL mirrors the effects observed for BOLD. Not surprisingly, the CBF-FC was generally lower than that of BOLD in both ASL implementations, in agreement with other studies comparing ASL and BOLD FC (Viviani et al., 2011; Jann et al., 2015a). This globally decreased FC strength is a consequence of intrinsically lower tSNR in ASL and due to the subtraction of label and control images that generates shorter timeseries for FC analysis in CBF data. However, while FC strength is lower, comparison of the spatial maps using DSC analysis revealed that the DMNs were similar between CBF and BOLD datasets. Similar to BOLD rs-fMRI, physiological noise reduction by using WM and CSF derived nuisance variables in 3D BS GRASE pCASL also resulted in improved spatial specificity when compared to a template DMN. Including head movement related nuisance variables into the preprocessing pipeline resulted in slight improvements of FC in anterior-posterior connections in 3D BS GRASE pCASL. This effect again was similar to the effect observed in the BOLD data (Van Dijk et al., 2010, 2012; Power et al., 2012, 2015). The voxel-wise repeated-measures ANOVA across NRSs confirmed the template-ROI based analyses between PCC and frontal areas and revealed additional areas in the inferior parietal lobes (IPL) where noise reduction had effects on FC. Voxel-wise maps were dominated by effects from physiological noise reduction that generally reduced FC in all DMN areas. Furthermore, the two frontal areas in 3D BS GRASE pCASL showed an increase in FC after motion correction although only the right mPFC ROI reached significance (Figure 4B1). The IPLs showed minor increases in FC whereas the PCC exhibited a minor reduction of local FC (Table 1). In 2D pCASL only the PCC was above the threshold for defining the DMN (compared to Figure 1). It showed significant decrease of local connectivity strength following motion regression in addition to physiological noise removal, and thus results are in agreement with the general observations of this study.

Finally, the whole brain parcellated connectivity analysis showed a similar motion related distance dependence of FC changes (Figure 5), with effects more pronounced for long-range than short-range connections. Notably, since this analysis was not limited to the DMN areas, the effect was observed in both the 3D BS GRASE and 2D dual-echo pCASL datasets. This suggests that although 2D pCASL shows low connectivity overall, on a less stringent threshold for connectivity results, it could still benefit from motion regression prior to FC analysis in the same fashion as the other datasets.

In summary, 3D BS GRASE pCASL revealed similar DMN maps at the same statistical threshold as BOLD, albeit with generally reduced FC strength. Moreover, 3D BS GRASE pCASL displayed similar FC changes as a function of different sets of nuisance variables used in the preprocessing, showing improved spatial specificity after physiological noise reduction and improved long-range connectivity with motion correction. In contrast, 2D dual-echo pCASL showed weak connectivity overall, which did not survive the same statistical threshold set for this study. This is most likely a problem of low tSNR for this ASL implementation that has no background suppression or the small sample size.

### Results for clinical cohort: children with ASD vs. matched TD children

In both ASD and TD groups, motion regression reduced local connectivity in posterior DMN areas while it significantly increased connectivity with superior frontal areas in the TD group and with orbitofrontal areas in the ASD group. Furthermore, in ASD we observed an increased anti-correlation between PCC and areas of the anterior salience network as well as areas of the somatomotor network. This suggests that noise reduction might affect not only within network effects (DMN), it may also benefit the separation between functional networks in autism. These alterations in FC due to noise reduction were also observed in direct group comparisons, leading to marked changes in observed group differences, both in terms of hyper- and hypoconnectivity. While all NRS (NRS 1,4,5) yielded altered connectivity in somatomotor network in ASD, NRS4&5 revealed reduced long-range FC from PCC to the dorsal portion of the prefrontal cortex and parahippocampal gyri, with suppression of hyperconnectivity with lateral-temporal areas. Overall, noise reduction altered the pattern of temporal lobe hyperconnectivity highlighting instead long-range hypoconnections to frontal areas and the medial temporal lobes (i.e., parahippocampal gyri). Similar motion related effects on FC were found in a study using independent component analysis to identify BOLD-DMN subnetworks in ASD (Starck et al., 2013). They reported that after accounting for motion effects, group differences between posterior and anterior DMN subnetworks, as well as in a ventral subnetwork including the parahippocampal gyrus were accentuated. Moreover, altered connectivity from PCC to superior frontal and the parahippocampal gyri in ASD have also been related to deficits in social functioning (Monk et al., 2009; Weng et al., 2010).

Recently, an anterior-posterior gradient of hyper- and hypoconnectivity received considerable attention in neuroimaging studies of ASD (Keown et al., 2013; Rudie and Dapretto, 2013; Di Martino et al., 2014) and is discussed in the context of improved selective cognitive abilities (local hyperconnectivity) and impaired social functioning (long-range hypoconnectivity between frontal and posterior cortices) (Jann et al., 2015b).

## Conclusion

Noise reduction affected FC analysis from a seed in the PCC to other brain areas of the DMN in all datasets (BOLD and pCASL). First, changes in FC strength and spatial maps of the DMN with regard to physiological nuisance variables (WM/CSF signals) and head movement related nuisance variables were replicated in two separate BOLD datasets, one with a conventional EPI implementation and another based on data acquired in a 2D dual-echo pCASL sequence. Second, analysis of NRS effects on FC analysis of CBF data demonstrated that 3D BS GRASE pCASL shows similar behavior as that observed for BOLD. The favorable noise properties of 3D BS GRASE pCASL as compared to 2D dual-echo pCASL and the improved tSNR after noise reduction render this pCASL implementation more suitable for CBF based FC analyses showing similar networks (Dai et al., 2015; Jann et al., 2015a) and dependence on noise reduction as BOLD. The dual-echo 2D pCASL used here provides perfusion and BOLD images with optimal contrasts, hence can provide proper BOLD based FC results and quantitative CBF (Zhu et al., 2013). FC analysis on the CBF timeseries of 2D pCASL should be treated with caution due to intrinsically low tSNRin conjunction with small sample size. However, a potential advantage of dual-echo ASL, that was not investigated here, is that the TE dependence of signal relaxation might be utilized to separate BOLD and non-BOLD signals (Kundu et al., 2012, 2013). The sensitivity of 2D pCASL can be improved in the future with optimized background suppression strategies in conjunction with multiband acquisitions (Shao et al., 2016).

Finally, applying the full spectrum of NRS in a cohort of children with ASD and typically developing controls we observed that 3D BS GRASE pCASL based FC analysis yielded results that are in accordance with effects of head motion and group differences between ASD and TD children observed in BOLD-DMNs. These findings underline the complex changes in functional organization in ASD and the impact that different preprocessing steps could have on the research findings (Nair et al., 2014).

## Author contributions

KJ designed the experiment, performed the analyses and wrote the manuscript. ER analyzed the data and RS designed the experiment and contributed analysis tools. MD wrote the manuscript. DW conceptualized the study and wrote the manuscript.

### Conflict of interest statement

The authors declare that the research was conducted in the absence of any commercial or financial relationships that could be construed as a potential conflict of interest.

## Supplementary material

Supplementary Table 1 **This table lists all *post-hoc t*-test (after ANOVA) *t* and *p*-values between all NRS in each brain area and for all 4 examined modalities**.

Supplementary Figure 1 **Differences in group wise FC seeded from the PCC after motion regression. (A)** Connectivity in Children with ASD for NRS4 and NRS5. **(B)** Difference between NRS4 and NRS5 in ASD. **(C)** Connectivity in TD children for NRS4 and NRS5. **(D)** Difference between NRS4 and NRS5 in TD.
