# Supplementary material for: Noise Reduction in Arterial Spin Labeling Based Functional Connectivity Using Nuisance Variables
Source: Front Neurosci. 2016 Aug 23;10:371. doi: 10.3389/fnins.2016.00371 (PMC4993769; doi:10.3389/fnins.2016.00371)
Supplement: Supplementary Table 1 — This table lists all post-hoc t-test (after ANOVA) t and p-values between all NRS in each brain area and for all 4 examined modalities. [file Table1.PDF]

3D BOLD

T-values

Region		NRS1	NRS2	NRS3	NRS4	NRS5
Fusiform_R	NRS1	-	1.68	2.20	20.63	9.24
	NRS2	-1.68	-	1.43	5.84	7.13
	NRS3	-2.20	-1.43	-	5.75	8.48
	NRS4	-20.63	-5.84	-5.75	-	0.40
	NRS5	-9.24	-7.13	-8.48	-0.40	-
Temporal_Mid_L	NRS1	-	-0.21	0.12	9.54	6.36
	NRS2	0.21	-	2.17	8.54	7.49
	NRS3	-0.12	-2.17	-	8.32	6.96
	NRS4	-9.54	-8.54	-8.32	-	0.01
	NRS5	-6.36	-7.49	-6.96	-0.01	-
Precuneus/ posterior cingulate	NRS1	-	2.27	2.97	14.25	10.54
	NRS2	-2.27	-	3.19	8.32	7.66
	NRS3	-2.97	-3.19	-	8.06	7.41
	NRS4	-14.25	-8.32	-8.06	-	1.20
	NRS5	-10.54	-7.66	-7.41	-1.20	-
Fusiform_L	NRS1	-	1.23	1.69	7.40	6.46
	NRS2	-1.23	-	1.38	4.56	6.50
	NRS3	-1.69	-1.38	-	4.43	6.87
	NRS4	-7.40	-4.56	-4.43	-	1.22
	NRS5	-6.46	-6.50	-6.87	-1.22	-
Cingulum_Ant_L	NRS1	-	-0.48	1.02	6.61	6.13
	NRS2	0.48	-	2.59	6.36	6.00
	NRS3	-1.02	-2.59	-	4.17	3.80
	NRS4	-6.61	-6.36	-4.17	-	-2.42
	NRS5	-6.13	-6.00	-3.80	2.42	-
Cingulum_Ant_R	NRS1	-	1.38	2.04	7.42	7.25
	NRS2	-1.38	-	1.72	6.61	6.89
	NRS3	-2.04	-1.72	-	4.75	5.00
	NRS4	-7.42	-6.61	-4.75	-	-0.08
	NRS5	-7.25	-6.89	-5.00	0.08	-
Occipital_Sup_R	NRS1	-	2.86	2.61	8.10	7.10
	NRS2	-2.86	-	0.40	4.25	4.08
	NRS3	-2.61	-0.40	-	4.56	4.41
	NRS4	-8.10	-4.25	-4.56	-	0.59
	NRS5	-7.10	-4.08	-4.41	-0.59	-
Parietal_Sup_L	NRS1	-	2.51	2.61	8.24	6.07
	NRS2	-2.51	-	1.11	4.31	4.17
	NRS3	-2.61	-1.11	-	3.93	3.82
	NRS4	-8.24	-4.31	-3.93	-	0.72
	NRS5	-6.07	-4.17	-3.82	-0.72	-

p-values

Region		NRS1	NRS2	NRS3	NRS4	NRS5
Fusiform_R	NRS1	-	0.1270	0.0558	0.0000	0.0000
	NRS2	0.1270	-	0.1876	0.0002	0.0001
	NRS3	0.0558	0.1876	-	0.0003	0.0000
	NRS4	0.0000	0.0002	0.0003	-	0.6996
	NRS5	0.0000	0.0001	0.0000	0.6996	-
Temporal_Mid_L	NRS1	-	0.8378	0.9042	0.0000	0.0001
	NRS2	0.8378	-	0.0582	0.0000	0.0000
	NRS3	0.9042	0.0582	-	0.0000	0.0001
	NRS4	0.0000	0.0000	0.0000	-	0.9912
	NRS5	0.0001	0.0000	0.0001	0.9912	-
Precuneus/ posterior cingulate	NRS1	-	0.0495	0.0156	0.0000	0.0000
	NRS2	0.0495	-	0.0111	0.0000	0.0000
	NRS3	0.0156	0.0111	-	0.0000	0.0000
	NRS4	0.0000	0.0000	0.0000	-	0.2598
	NRS5	0.0000	0.0000	0.0000	0.2598	-
Fusiform_L	NRS1	-	0.2488	0.1247	0.0000	0.0001
	NRS2	0.2488	-	0.2008	0.0014	0.0001
	NRS3	0.1247	0.2008	-	0.0016	0.0001
	NRS4	0.0000	0.0014	0.0016	-	0.2534
	NRS5	0.0001	0.0001	0.0001	0.2534	-
Cingulum_Ant_L	NRS1	-	0.6404	0.3329	0.0001	0.0002
	NRS2	0.6404	-	0.0292	0.0001	0.0002
	NRS3	0.3329	0.0292	-	0.0024	0.0042
	NRS4	0.0001	0.0001	0.0024	-	0.0386
	NRS5	0.0002	0.0002	0.0042	0.0386	-
Cingulum_Ant_R	NRS1	-	0.2015	0.0713	0.0000	0.0000
	NRS2	0.2015	-	0.1191	0.0001	0.0001
	NRS3	0.0713	0.1191	-	0.0010	0.0007
	NRS4	0.0000	0.0001	0.0010	-	0.9390
	NRS5	0.0000	0.0001	0.0007	0.9390	-
Occipital_Sup_R	NRS1	-	0.0188	0.0282	0.0000	0.0001
	NRS2	0.0188	-	0.6992	0.0021	0.0027
	NRS3	0.0282	0.6992	-	0.0014	0.0017
	NRS4	0.0000	0.0021	0.0014	-	0.5716
	NRS5	0.0001	0.0027	0.0017	0.5716	-
Parietal_Sup_L	NRS1	-	0.0332	0.0282	0.0000	0.0002
	NRS2	0.0332	-	0.2967	0.0020	0.0024
	NRS3	0.0282	0.2967	-	0.0035	0.0041
	NRS4	0.0000	0.0020	0.0035	-	0.4925
	NRS5	0.0002	0.0024	0.0041	0.4925	-

3D ASL

T-values and **p-values**

Frontal_Sup_Medial_R

T-values

	NRS1	NRS2	NRS3	NRS4	NRS5
**NRS1**	-	1.24	0.98	3.98	3.70
**NRS2**	-1.24	-	-0.91	4.50	5.12
**NRS3**	-0.98	0.91	-	5.31	5.98
**NRS4**	-3.98	-4.50	-5.31	-	-0.54
**NRS5**	-3.70	-5.12	-5.98	0.54	-

p-values

	NRS1	NRS2	NRS3	NRS4	NRS5
**NRS1**	-	0.2453	0.3509	0.0032	0.0050
**NRS2**	0.2453	-	0.3882	0.0015	0.0006
**NRS3**	0.3509	0.3882	-	0.0005	0.0002
**NRS4**	0.0032	0.0015	0.0005	-	0.6032
**NRS5**	0.0050	0.0006	0.0002	0.6032	-

Cingulum_Ant_L

T-values

	NRS1	NRS2	NRS3	NRS4	NRS5
**NRS1**	-	0.02	0.02	5.35	3.80
**NRS2**	-0.02	-	0.02	7.50	6.05
**NRS3**	-0.02	-0.02	-	7.27	6.14
**NRS4**	-5.35	-7.50	-7.27	-	-3.34
**NRS5**	-3.80	-6.05	-6.14	3.34	-

p-values

	NRS1	NRS2	NRS3	NRS4	NRS5
**NRS1**	-	0.9855	0.9831	0.0005	0.0042
**NRS2**	0.9855	-	0.9819	0.0000	0.0002
**NRS3**	0.9831	0.9819	-	0.0000	0.0002
**NRS4**	0.0005	0.0000	0.0000	-	0.0087
**NRS5**	0.0042	0.0002	0.0002	0.0087	-

Precuneus/ posterior cingulate

T-values

	NRS1	NRS2	NRS3	NRS4	NRS5
**NRS1**	-	0.92	0.76	5.36	5.43
**NRS2**	-0.92	-	-0.68	5.24	5.31
**NRS3**	-0.76	0.68	-	5.68	5.83
**NRS4**	-5.36	-5.24	-5.68	-	0.61
**NRS5**	-5.43	-5.31	-5.83	-0.61	-

p-values

	NRS1	NRS2	NRS3	NRS4	NRS5
**NRS1**	-	0.3797	0.4695	0.0005	0.0004
**NRS2**	0.3797	-	0.5158	0.0005	0.0005
**NRS3**	0.4695	0.5158	-	0.0003	0.0002
**NRS4**	0.0005	0.0005	0.0003	-	0.5562
**NRS5**	0.0004	0.0005	0.0002	0.5562	-

SupraMarginal_R

T-values

	NRS1	NRS2	NRS3	NRS4	NRS5
**NRS1**	-	1.38	1.18	5.19	4.16
**NRS2**	-1.38	-	-0.34	4.80	4.55
**NRS3**	-1.18	0.34	-	5.64	5.46
**NRS4**	-5.19	-4.80	-5.64	-	-0.11
**NRS5**	-4.16	-4.55	-5.46	0.11	-

p-values

	NRS1	NRS2	NRS3	NRS4	NRS5
**NRS1**	-	0.2012	0.2690	0.0006	0.0024
**NRS2**	0.2012	-	0.7399	0.0010	0.0014
**NRS3**	0.2690	0.7399	-	0.0003	0.0004
**NRS4**	0.0006	0.0010	0.0003	-	0.9122
**NRS5**	0.0024	0.0014	0.0004	0.9122	-

sub lobar

T-values

	NRS1	NRS2	NRS3	NRS4	NRS5
**NRS1**	-	0.56	0.53	5.28	5.35
**NRS2**	-0.56	-	0.07	5.07	5.25
**NRS3**	-0.53	-0.07	-	4.71	5.34
**NRS4**	-5.28	-5.07	-4.71	-	0.05
**NRS5**	-5.35	-5.25	-5.34	-0.05	-

p-values

	NRS1	NRS2	NRS3	NRS4	NRS5
**NRS1**	-	0.5919	0.6074	0.0005	0.0005
**NRS2**	0.5919	-	0.9443	0.0007	0.0005
**NRS3**	0.6074	0.9443	-	0.0011	0.0005
**NRS4**	0.0005	0.0007	0.0011	-	0.9604
**NRS5**	0.0005	0.0005	0.0005	0.9604	-

SupraMarginal_L

T-values

	NRS1	NRS2	NRS3	NRS4	NRS5
**NRS1**	-	0.20	-0.05	5.74	3.21
**NRS2**	-0.20	-	-0.76	4.78	3.44
**NRS3**	0.05	0.76	-	5.88	4.23
**NRS4**	-5.74	-4.78	-5.88	-	-2.00
**NRS5**	-3.21	-3.44	-4.23	2.00	-

p-values

	NRS1	NRS2	NRS3	NRS4	NRS5
**NRS1**	-	0.8494	0.9593	0.0003	0.0107
**NRS2**	0.8494	-	0.4653	0.0010	0.0074
**NRS3**	0.9593	0.4653	-	0.0002	0.0022
**NRS4**	0.0003	0.0010	0.0002	-	0.0770
**NRS5**	0.0107	0.0074	0.0022	0.0770	-

2D BOLD

Temporal_Mid_L

	T-values NRS1	NRS2	NRS3	NRS4	NRS5	p-values NRS1	NRS2	NRS3	NRS4	NRS5
NRS1	-	-3.02	-3.01	4.30	3.06	-	0.0145	0.0148	0.0020	0.0136
NRS2	3.02	-	0.31	5.90	5.45	0.0145	-	0.7630	0.0002	0.0004
NRS3	3.01	-0.31	-	5.85	5.44	0.0148	0.7630	-	0.0002	0.0004
NRS4	-4.30	-5.90	-5.85	-	-1.35	0.0020	0.0002	0.0002	-	0.2113
NRS5	-3.06	-5.45	-5.44	1.35	-	0.0136	0.0004	0.0004	0.2113	-

Frontal_Inf_Orb_L

	T-values NRS1	NRS2	NRS3	NRS4	NRS5	p-values NRS1	NRS2	NRS3	NRS4	NRS5
NRS1	-	-1.48	-1.45	7.36	4.58	-	0.1738	0.1800	0.0000	0.0013
NRS2	1.48	-	-0.24	6.39	8.02	0.1738	-	0.8187	0.0001	0.0000
NRS3	1.45	0.24	-	6.25	7.68	0.1800	0.8187	-	0.0001	0.0000
NRS4	-7.36	-6.39	-6.25	-	-0.13	0.0000	0.0001	0.0001	-	0.8987
NRS5	-4.58	-8.02	-7.68	0.13	-	0.0013	0.0000	0.0000	0.8987	-

Cingulum_Ant_L

	T-values NRS1	NRS2	NRS3	NRS4	NRS5	p-values NRS1	NRS2	NRS3	NRS4	NRS5
NRS1	-	-1.78	-1.65	2.56	2.29	-	0.1091	0.1332	0.0306	0.0481
NRS2	1.78	-	1.65	4.52	4.04	0.1091	-	0.1341	0.0014	0.0029
NRS3	1.65	-1.65	-	4.37	3.95	0.1332	0.1341	-	0.0018	0.0034
NRS4	-2.56	-4.52	-4.37	-	-0.59	0.0306	0.0014	0.0018	-	0.5717
NRS5	-2.29	-4.04	-3.95	0.59	-	0.0481	0.0029	0.0034	0.5717	-

Cingulum_Mid_R

	T-values NRS1	NRS2	NRS3	NRS4	NRS5	p-values NRS1	NRS2	NRS3	NRS4	NRS5
NRS1	-	-0.06	0.23	10.49	10.59	-	0.9572	0.8265	0.0000	0.0000
NRS2	0.06	-	2.80	9.81	10.13	0.9572	-	0.0206	0.0000	0.0000
NRS3	-0.23	-2.80	-	9.68	9.98	0.8265	0.0206	-	0.0000	0.0000
NRS4	-10.49	-9.81	-9.68	-	1.46	0.0000	0.0000	0.0000	-	0.1791
NRS5	-10.59	-10.13	-9.98	-1.46	-	0.0000	0.0000	0.0000	0.1791	-

Cingulum_Ant_R

	T-values NRS1	NRS2	NRS3	NRS4	NRS5	p-values NRS1	NRS2	NRS3	NRS4	NRS5
NRS1	-	-2.31	-2.14	4.11	3.44	-	0.0462	0.0610	0.0026	0.0074
NRS2	2.31	-	1.76	6.57	7.28	0.0462	-	0.1114	0.0001	0.0000
NRS3	2.14	-1.76	-	6.24	6.94	0.0610	0.1114	-	0.0002	0.0001
NRS4	-4.11	-6.57	-6.24	-	-1.56	0.0026	0.0001	0.0002	-	0.1521
NRS5	-3.44	-7.28	-6.94	1.56	-	0.0074	0.0000	0.0001	0.1521	-

Angular_R

	T-values NRS1	NRS2	NRS3	NRS4	NRS5	p-values NRS1	NRS2	NRS3	NRS4	NRS5
NRS1	-	-2.60	-2.51	6.69	5.40	-	0.0288	0.0332	0.0001	0.0004
NRS2	2.60	-	1.88	9.83	9.08	0.0288	-	0.0924	0.0000	0.0000
NRS3	2.51	-1.88	-	9.75	8.98	0.0332	0.0924	-	0.0000	0.0000
NRS4	-6.69	-9.83	-9.75	-	-2.64	0.0001	0.0000	0.0000	-	0.0268
NRS5	-5.40	-9.08	-8.98	2.64	-	0.0004	0.0000	0.0000	0.0268	-

Angular_L

	T-values NRS1	NRS2	NRS3	NRS4	NRS5	p-values NRS1	NRS2	NRS3	NRS4	NRS5
NRS1	-	-2.08	-1.89	4.51	4.86	-	0.0678	0.0920	0.0015	0.0009
NRS2	2.08	-	1.39	6.61	7.97	0.0678	-	0.1973	0.0001	0.0000
NRS3	1.89	-1.39	-	6.70	8.14	0.0920	0.1973	-	0.0001	0.0000
NRS4	-4.51	-6.61	-6.70	-	-0.99	0.0015	0.0001	0.0001	-	0.3491
NRS5	-4.86	-7.97	-8.14	0.99	-	0.0009	0.0000	0.0000	0.3491	-

Frontal_Mid_L

	T-values NRS1	NRS2	NRS3	NRS4	NRS5	p-values NRS1	NRS2	NRS3	NRS4	NRS5
NRS1	-	-1.72	-1.47	6.56	6.68	-	0.1203	0.1745	0.0001	0.0001
NRS2	1.72	-	1.73	8.66	8.61	0.1203	-	0.1168	0.0000	0.0000
NRS3	1.47	-1.73	-	8.64	8.66	0.1745	0.1168	-	0.0000	0.0000
NRS4	-6.56	-8.66	-8.64	-	0.15	0.0001	0.0000	0.0000	-	0.8875
NRS5	-6.68	-8.61	-8.66	-0.15	-	0.0001	0.0000	0.0000	0.8875	-

Frontal_Sup_R

	T-values NRS1	NRS2	NRS3	NRS4	NRS5	p-values NRS1	NRS2	NRS3	NRS4	NRS5
NRS1	-	-2.90	-2.67	5.06	4.03	-	0.0175	0.0255	0.0007	0.0030
NRS2	2.90	-	2.88	6.80	8.42	0.0175	-	0.0183	0.0001	0.0000
NRS3	2.67	-2.88	-	6.51	7.99	0.0255	0.0183	-	0.0001	0.0000
NRS4	-5.06	-6.80	-6.51	-	-2.03	0.0007	0.0001	0.0001	-	0.0735
NRS5	-4.03	-8.42	-7.99	2.03	-	0.0030	0.0000	0.0000	0.0735	-

Paracentral_Lobule_R

	T-values NRS1	NRS2	NRS3	NRS4	NRS5	p-values NRS1	NRS2	NRS3	NRS4	NRS5
NRS1	-	-0.68	-0.47	8.58	7.94	-	0.5151	0.6526	0.0000	0.0000
NRS2	0.68	-	2.19	13.42	12.19	0.5151	-	0.0559	0.0000	0.0000
NRS3	0.47	-2.19	-	13.22	11.95	0.6526	0.0559	-	0.0000	0.0000
NRS4	-8.58	-13.42	-13.22	-	2.46	0.0000	0.0000	0.0000	-	0.0363
NRS5	-7.94	-12.19	-11.95	-2.46	-	0.0000	0.0000	0.0000	0.0363	-

2D ASL

T-values

Posterior Cingulate	NRS1	NRS2	NRS3	NRS4	NRS5
**NRS1**	-	-1.31	-0.50	4.30	6.39
**NRS2**	1.31	-	1.03	4.85	7.22
**NRS3**	0.50	-1.03	-	4.75	7.28
**NRS4**	-4.30	-4.85	-4.75	-	3.26
**NRS5**	-6.39	-7.22	-7.28	-3.26	-

p-values

	NRS1	NRS2	NRS3	NRS4	NRS5
**NRS1**	-	0.2241	0.6260	0.0020	0.0001
**NRS2**	0.2241	-	0.3288	0.0009	0.0000
**NRS3**	0.6260	0.3288	-	0.0010	0.0000
**NRS4**	0.0020	0.0009	0.0010	-	0.0099
**NRS5**	0.0001	0.0000	0.0000	0.0099	-
