# Supplementary figures and images for: Noise Reduction in Arterial Spin Labeling Based Functional Connectivity Using Nuisance Variables
Source: Front Neurosci. 2016 Aug 23;10:371. doi: 10.3389/fnins.2016.00371 (PMC4993769; doi:10.3389/fnins.2016.00371)

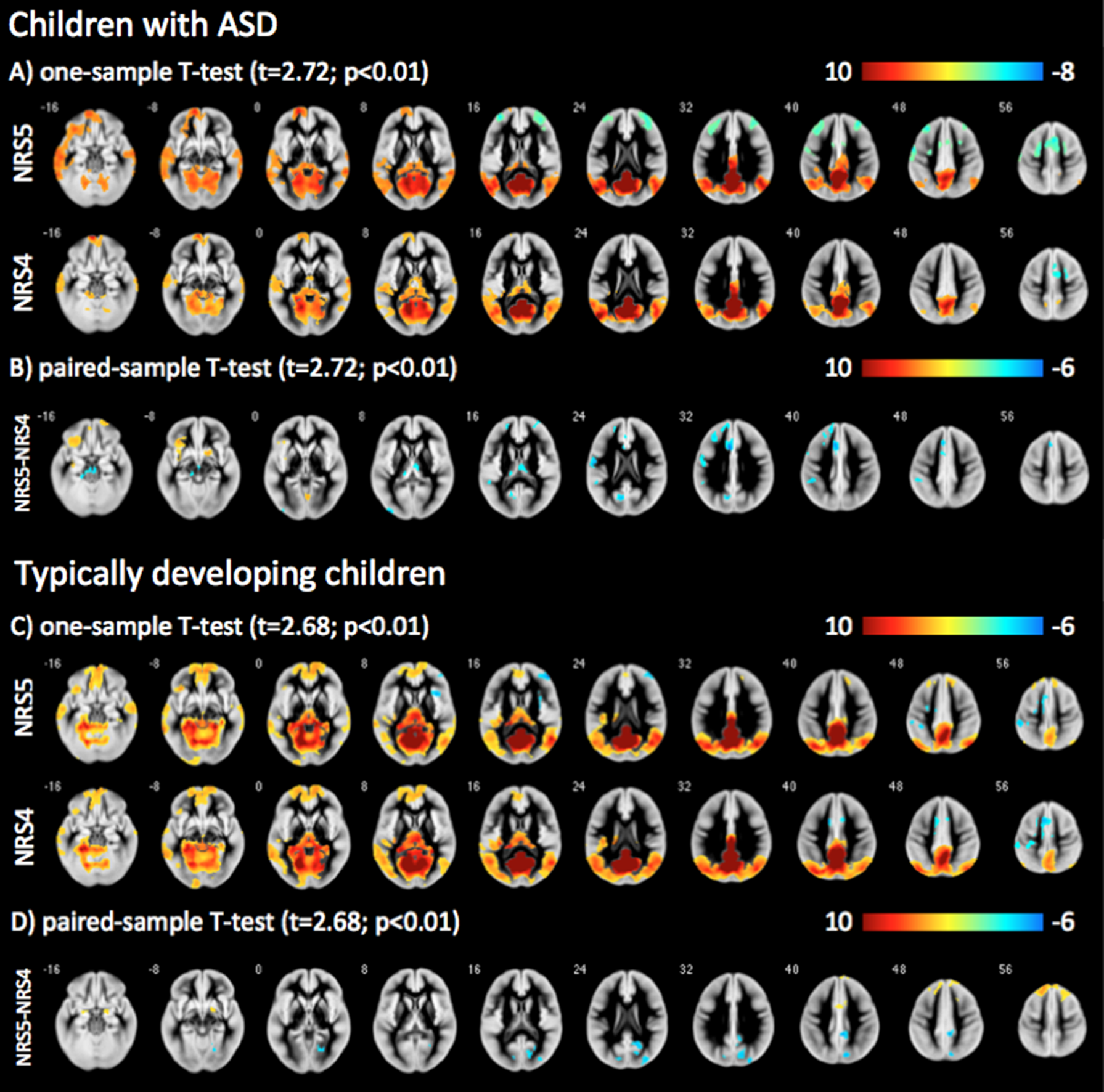

Supplement: Supplementary Figure 1 — Differences in group wise FC seeded from the PCC after motion regression. (A) Connectivity in Children with ASD for NRS4 and NRS5. (B) Difference between NRS4 and NRS5 in ASD. (C) Connectivity in TD children for NRS4 and NRS5. (D) Difference between NRS4 and NRS5 in TD. [file Image1.TIF]
